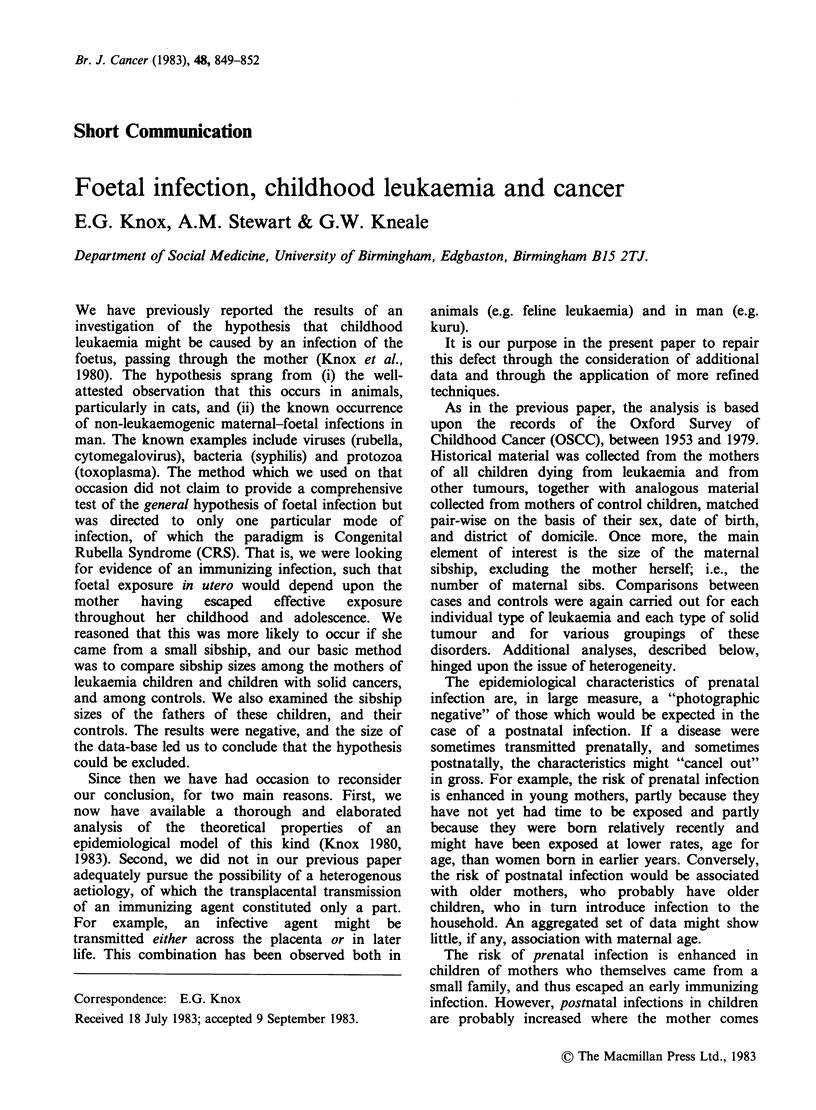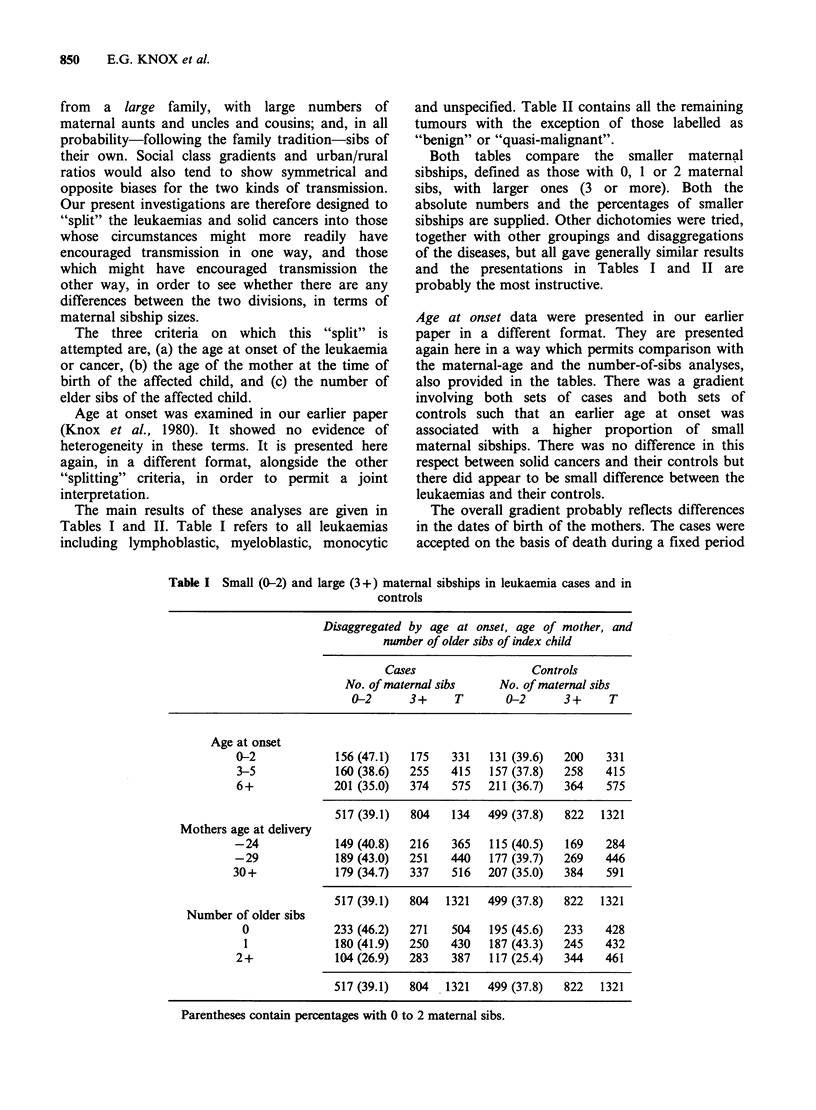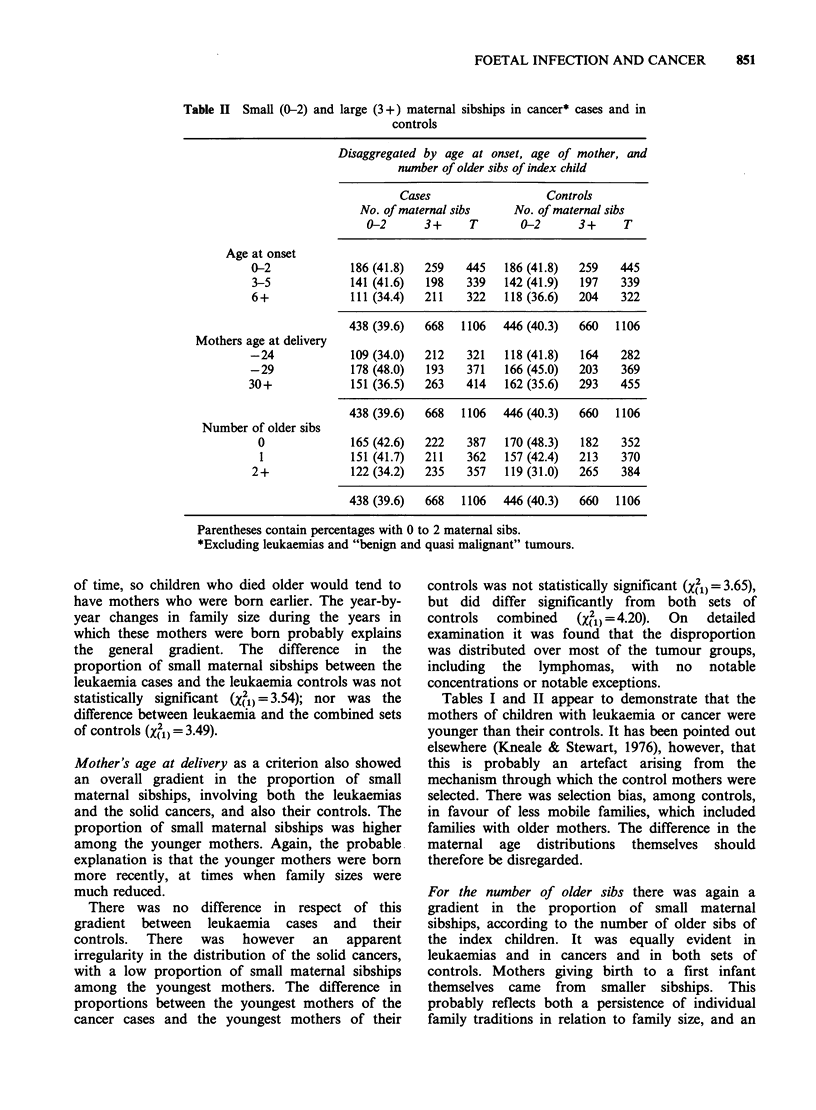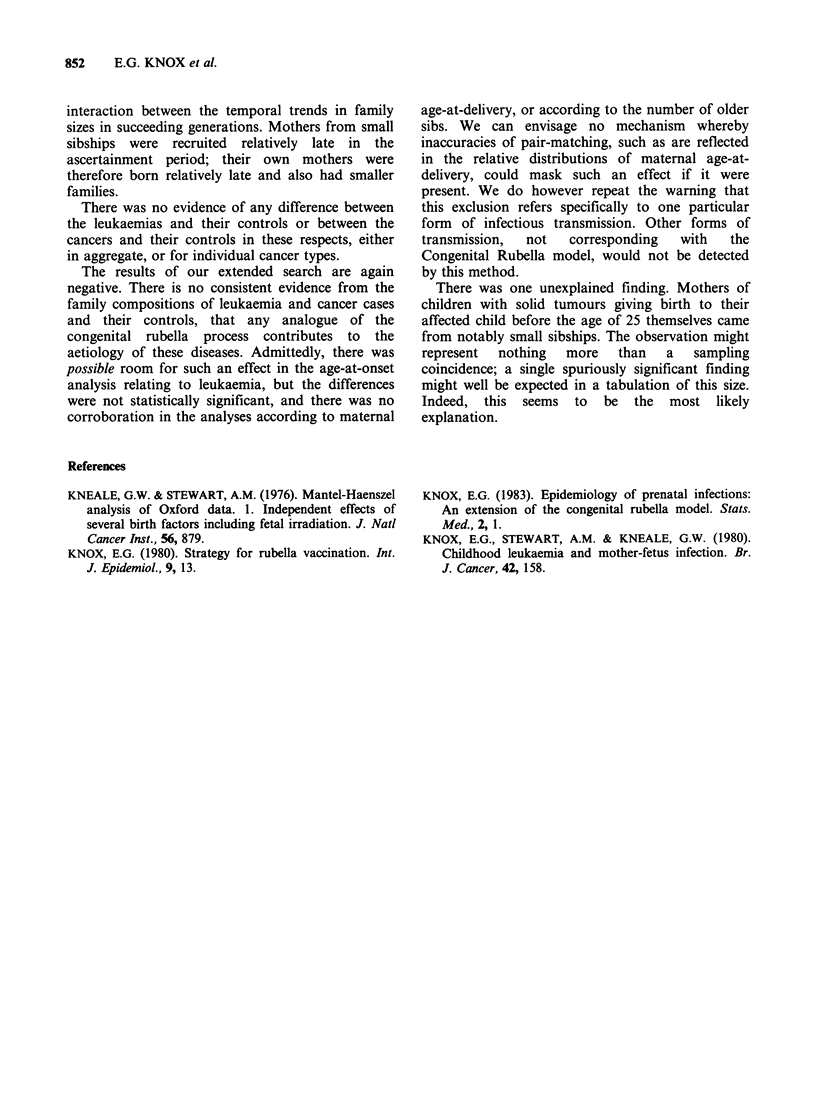# Foetal infection, childhood leukaemia and cancer.

**DOI:** 10.1038/bjc.1983.276

**Published:** 1983-12

**Authors:** E. G. Knox, A. M. Stewart, G. W. Kneale


Br. J. Cancer (1983), 48, 849–852

### Short Communication

### Foetal infection, childhood leukaemia and cancer

E.G. Knox, A.M. Stewart & G.W. Kneale

*Department of Social Medicine, University of Birmingham, Edgbaston, Birmingham B15 2TJ.*

We have previously reported the results of an investigation of the hypothesis that childhood leukaemia might be caused by an infection of the foetus, passing through the mother (Knox *et al.*, 1980). The hypothesis sprang from (i) the well-attested observation that this occurs in animals, particularly in cats, and (ii) the known occurrence of non-leukaemogenic maternal–foetal infections in man. The known examples include viruses (rubella, cytomegalovirus), bacteria (syphilis) and protozoa (toxoplasma). The method which we used on that occasion did not claim to provide a comprehensive test of the *general* hypothesis of foetal infection but was directed to only one particular mode of infection, of which the paradigm is Congenital Rubella Syndrome (CRS). That is, we were looking for evidence of an immunizing infection, such that foetal exposure *in utero* would depend upon the mother having escaped effective exposure throughout her childhood and adolescence. We reasoned that this was more likely to occur if she came from a small sibship, and our basic method was to compare sibship sizes among the mothers of leukaemia children and children with solid cancers, and among controls. We also examined the sibship sizes of the fathers of these children, and their controls. The results were negative, and the size of the data-base led us to conclude that the hypothesis could be excluded.

Since then we have had occasion to reconsider our conclusion, for two main reasons. First, we now have available a thorough and elaborated analysis of the theoretical properties of an epidemiological model of this kind (Knox 1980, 1983). Second, we did not in our previous paper adequately pursue the possibility of a heterogenous aetiology, of which the transplacental transmission of an immunizing agent constituted only a part. For example, an infective agent might be transmitted *either* across the placenta *or* in later life. This combination has been observed both in animals (e.g. feline leukaemia) and in man (e.g. kuru).

It is our purpose in the present paper to repair this defect through the consideration of additional data and through the application of more refined techniques.

As in the previous paper, the analysis is based upon the records of the Oxford Survey of Childhood Cancer (OSCC), between 1953 and 1979. Historical material was collected from the mothers of all children dying from leukaemia and from other tumours, together with analogous material collected from mothers of control children, matched pair-wise on the basis of their sex, date of birth, and district of domicile. Once more, the main element of interest is the size of the maternal sibship, excluding the mother herself; i.e., the number of maternal sibs. Comparisons between cases and controls were again carried out for each individual type of leukaemia and each type of solid tumour and for various groupings of these disorders. Additional analyses, described below, hinged upon the issue of heterogeneity.

The epidemiological characteristics of prenatal infection are, in large measure, a "photographic negative" of those which would be expected in the case of a postnatal infection. If a disease were sometimes transmitted prenatally, and sometimes postnatally, the characteristics might "cancel out" in gross. For example, the risk of prenatal infection is enhanced in young mothers, partly because they have not yet had time to be exposed and partly because they were born relatively recently and might have been exposed at lower rates, age for age, than women born in earlier years. Conversely, the risk of postnatal infection would be associated with older mothers, who probably have older children, who in turn introduce infection to the household. An aggregated set of data might show little, if any, association with maternal age.

The risk of *pre*natal infection is enhanced in children of mothers who themselves came from a small family, and thus escaped an early immunizing infection. However, *post*natal infections in children are probably increased where the mother comes

Correspondence: E.G. Knox

Received 18 July 1983; accepted 9 September 1983.

© The Macmillan Press Ltd., 1983

from a *large* family, with large numbers of maternal aunts and uncles and cousins; and, in all probability—following the family tradition—sibs of their own. Social class gradients and urban/rural ratios would also tend to show symmetrical and opposite biases for the two kinds of transmission. Our present investigations are therefore designed to "split" the leukaemias and solid cancers into those whose circumstances might more readily have encouraged transmission in one way, and those which might have encouraged transmission the other way, in order to see whether there are any differences between the two divisions, in terms of maternal sibship sizes.

The three criteria on which this "split" is attempted are, (a) the age at onset of the leukaemia or cancer, (b) the age of the mother at the time of birth of the affected child, and (c) the number of elder sibs of the affected child.

Age at onset was examined in our earlier paper (Knox *et al.*, 1980). It showed no evidence of heterogeneity in these terms. It is presented here again, in a different format, alongside the other "splitting" criteria, in order to permit a joint interpretation.

The main results of these analyses are given in Tables I and II. Table I refers to all leukaemias including lymphoblastic, myeloblastic, monocytic and unspecified. Table II contains all the remaining tumours with the exception of those labelled as "benign" or "quasi-malignant".

Both tables compare the smaller maternal sibships, defined as those with 0, 1 or 2 maternal sibs, with larger ones (3 or more). Both the absolute numbers and the percentages of smaller sibships are supplied. Other dichotomies were tried, together with other groupings and disaggregations of the diseases, but all gave generally similar results and the presentations in Tables I and II are probably the most instructive.

*Age at onset* data were presented in our earlier paper in a different format. They are presented again here in a way which permits comparison with the maternal-age and the number-of-sibs analyses, also provided in the tables. There was a gradient involving both sets of cases and both sets of controls such that an earlier age at onset was associated with a higher proportion of small maternal sibships. There was no difference in this respect between solid cancers and their controls but there did appear to be small difference between the leukaemias and their controls.

The overall gradient probably reflects differences in the dates of birth of the mothers. The cases were accepted on the basis of death during a fixed period

**Table I** Small (0–2) and large (3+) maternal sibships in leukaemia cases and in controls

| | *Disaggregated by age at onset, age of mother, and number of older sibs of index child* | | | | | |
|---|---|---|---|---|---|---|
| | *Cases* | | | *Controls* | | |
| | *No. of maternal sibs* | | | *No. of maternal sibs* | | |
| | *0–2* | *3+* | *T* | *0–2* | *3+* | *T* |
| Age at onset | | | | | | |
| 0–2 | 156 (47.1) | 175 | 331 | 131 (39.6) | 200 | 331 |
| 3–5 | 160 (38.6) | 255 | 415 | 157 (37.8) | 258 | 415 |
| 6+ | 201 (35.0) | 374 | 575 | 211 (36.7) | 364 | 575 |
| | 517 (39.1) | 804 | 134 | 499 (37.8) | 822 | 1321 |
| Mothers age at delivery | | | | | | |
| −24 | 149 (40.8) | 216 | 365 | 115 (40.5) | 169 | 284 |
| −29 | 189 (43.0) | 251 | 440 | 177 (39.7) | 269 | 446 |
| 30+ | 179 (34.7) | 337 | 516 | 207 (35.0) | 384 | 591 |
| | 517 (39.1) | 804 | 1321 | 499 (37.8) | 822 | 1321 |
| Number of older sibs | | | | | | |
| 0 | 233 (46.2) | 271 | 504 | 195 (45.6) | 233 | 428 |
| 1 | 180 (41.9) | 250 | 430 | 187 (43.3) | 245 | 432 |
| 2+ | 104 (26.9) | 283 | 387 | 117 (25.4) | 344 | 461 |
| | 517 (39.1) | 804 | 1321 | 499 (37.8) | 822 | 1321 |

Parentheses contain percentages with 0 to 2 maternal sibs.

**Table II** Small (0–2) and large (3+) maternal sibships in cancer* cases and in controls

| | *Disaggregated by age at onset, age of mother, and number of older sibs of index child* | | | | | |
|---|---|---|---|---|---|---|
| | *Cases* | | | *Controls* | | |
| | *No. of maternal sibs* | | | *No. of maternal sibs* | | |
| | *0–2* | *3+* | *T* | *0–2* | *3+* | *T* |
| Age at onset | | | | | | |
| 0–2 | 186 (41.8) | 259 | 445 | 186 (41.8) | 259 | 445 |
| 3–5 | 141 (41.6) | 198 | 339 | 142 (41.9) | 197 | 339 |
| 6+ | 111 (34.4) | 211 | 322 | 118 (36.6) | 204 | 322 |
| | 438 (39.6) | 668 | 1106 | 446 (40.3) | 660 | 1106 |
| Mothers age at delivery | | | | | | |
| −24 | 109 (34.0) | 212 | 321 | 118 (41.8) | 164 | 282 |
| −29 | 178 (48.0) | 193 | 371 | 166 (45.0) | 203 | 369 |
| 30+ | 151 (36.5) | 263 | 414 | 162 (35.6) | 293 | 455 |
| | 438 (39.6) | 668 | 1106 | 446 (40.3) | 660 | 1106 |
| Number of older sibs | | | | | | |
| 0 | 165 (42.6) | 222 | 387 | 170 (48.3) | 182 | 352 |
| 1 | 151 (41.7) | 211 | 362 | 157 (42.4) | 213 | 370 |
| 2+ | 122 (34.2) | 235 | 357 | 119 (31.0) | 265 | 384 |
| | 438 (39.6) | 668 | 1106 | 446 (40.3) | 660 | 1106 |

Parentheses contain percentages with 0 to 2 maternal sibs.
*Excluding leukaemias and "benign and quasi malignant" tumours.

of time, so children who died older would tend to have mothers who were born earlier. The year-by-year changes in family size during the years in which these mothers were born probably explains the general gradient. The difference in the proportion of small maternal sibships between the leukaemia cases and the leukaemia controls was not statistically significant ($\chi^2_{(1)}=3.54$); nor was the difference between leukaemia and the combined sets of controls ($\chi^2_{(1)}=3.49$).

*Mother's age at delivery* as a criterion also showed an overall gradient in the proportion of small maternal sibships, involving both the leukaemias and the solid cancers, and also their controls. The proportion of small maternal sibships was higher among the younger mothers. Again, the probable explanation is that the younger mothers were born more recently, at times when family sizes were much reduced.

There was no difference in respect of this gradient between leukaemia cases and their controls. There was however an apparent irregularity in the distribution of the solid cancers, with a low proportion of small maternal sibships among the youngest mothers. The difference in proportions between the youngest mothers of the cancer cases and the youngest mothers of their controls was not statistically significant ($\chi^2_{(1)}=3.65$), but did differ significantly from both sets of controls combined ($\chi^2_{(1)}=4.20$). On detailed examination it was found that the disproportion was distributed over most of the tumour groups, including the lymphomas, with no notable concentrations or notable exceptions.

Tables I and II appear to demonstrate that the mothers of children with leukaemia or cancer were younger than their controls. It has been pointed out elsewhere (Kneale & Stewart, 1976), however, that this is probably an artefact arising from the mechanism through which the control mothers were selected. There was selection bias, among controls, in favour of less mobile families, which included families with older mothers. The difference in the maternal age distributions themselves should therefore be disregarded.

*For the number of older sibs* there was again a gradient in the proportion of small maternal sibships, according to the number of older sibs of the index children. It was equally evident in leukaemias and in cancers and in both sets of controls. Mothers giving birth to a first infant themselves came from smaller sibships. This probably reflects both a persistence of individual family traditions in relation to family size, and an interaction between the temporal trends in family sizes in succeeding generations. Mothers from small sibships were recruited relatively late in the ascertainment period; their own mothers were therefore born relatively late and also had smaller families.

There was no evidence of any difference between the leukaemias and their controls or between the cancers and their controls in these respects, either in aggregate, or for individual cancer types.

The results of our extended search are again negative. There is no consistent evidence from the family compositions of leukaemia and cancer cases and their controls, that any analogue of the congenital rubella process contributes to the aetiology of these diseases. Admittedly, there was *possible* room for such an effect in the age-at-onset analysis relating to leukaemia, but the differences were not statistically significant, and there was no corroboration in the analyses according to maternal age-at-delivery, or according to the number of older sibs. We can envisage no mechanism whereby inaccuracies of pair-matching, such as are reflected in the relative distributions of maternal age-at-delivery, could mask such an effect if it were present. We do however repeat the warning that this exclusion refers specifically to one particular form of infectious transmission. Other forms of transmission, not corresponding with the Congenital Rubella model, would not be detected by this method.

There was one unexplained finding. Mothers of children with solid tumours giving birth to their affected child before the age of 25 themselves came from notably small sibships. The observation might represent nothing more than a sampling coincidence; a single spuriously significant finding might well be expected in a tabulation of this size. Indeed, this seems to be the most likely explanation.

### References

KNEALE, G.W. & STEWART, A.M. (1976). Mantel-Haenszel analysis of Oxford data. 1. Independent effects of several birth factors including fetal irradiation. *J. Natl Cancer Inst.*, **56**, 879.

KNOX, E.G. (1980). Strategy for rubella vaccination. *Int. J. Epidemiol.*, **9**, 13.

KNOX, E.G. (1983). Epidemiology of prenatal infections: An extension of the congenital rubella model. *Stats. Med.*, **2**, 1.

KNOX, E.G., STEWART, A.M. & KNEALE, G.W. (1980). Childhood leukaemia and mother-fetus infection. *Br. J. Cancer*, **42**, 158.